# Expression of ERCC1, Bcl-2, Lin28a, and Ki-67 as biomarkers of response to first-line platinum-based chemotherapy in patients with high-grade extrapulmonary neuroendocrine carcinomas or small cell lung cancer

**DOI:** 10.3332/ecancer.2017.767

**Published:** 2017-09-11

**Authors:** Juliana Florinda de M Rêgo, Raphael Salles Scortegagna de Medeiros, Maria Ignez Braghiroli, Breno Galvão, João Evangelista Bezerra Neto, Rodrigo Ramella Munhoz, Juliana Guerra, Suely Nonogaki, Lidia Kimura, Tulio Eduardo Pfiffer, Gilberto de Castro, Paulo Marcelo Hoff, Duilio Rocha Filho, Frederico Perego Costa, Rachel P Riechelmann

**Affiliations:** 1 Instituto do Cancer do Estado de São Paulo - Adress: Dr Arnaldo Av, 251 - Sao Paulo/SP, 01246-000, Brazil; 2 Universidade Federal do Rio Grande do Norte - Adress: Nilo Peçanha Av, 620 - Natal/RN, 59012-300, Brazil; 3 Liga NorteRiograndense Contra o Cancer - Adress: Miguel Castro Av, 1355 - Natal/RN, 59075-740, Brazil; 4 Universidade de Sao Paulo - Adress: Dr Ovídio Pires de Campos St, 225 - Sao Paulo/SP, 05403-010, Brazil; 5 Hospital Sirio Libanês - Adress: Dona Adma Jafet St, 115 – Sao Paulo/SP, 01308-050, Brazil; 6 Instituto Adolfo Lutz - Adress: Dr Arnaldo Av, 355 - Sao Paulo/SP, 01246-000, Brazil; 7 Instituto do Cancer do Ceara - Adress: Papi Júnior St - Fortaleza/CE, 60351-010, Brazil

**Keywords:** small cell lung cancer, extrapulmonary neuroendocrine carcinoma, biomarkers

## Abstract

**Background:**

Small cell lung cancer (SCLC) and high-grade extrapulmonary neuroendocrine carcinomas (EPNEC) share similar histopathological features and treatment, but outcomes may differ. We evaluated in our study the expression of biomarkers associated with response rate (RR) to chemotherapy and overall survival (OS) for these entities.

**Materials and Methods:**

This is a multicentre retrospective analysis of advanced EPNEC and SCLC patients treated with platinum-based chemotherapy. Paraffin-embedded tumour samples were reviewed by a single pathologist and tested for immunohistochemistry (IHC) expression of Ki-67, ERCC1, Bcl-2, and Lin28a. All images were evaluated by the same radiologist and RR was determined by RECIST 1.1.

**Results:**

From July, 2006 to July, 2014, 142 patients were identified, being 82 (57.7%) SCLC and 60 (42.3%) EPNEC. Clinical characteristics and median Ki-67 (SCLC: 60%; EPNEC: 50%; p = 0.86) were similar between the groups. RR was higher for SCLC patients (86.8% versus 44.6%; p<0.001), but median OS was similar (10.3 months in SCLC and 11.1 months in EPNEC; HR 0.69, p = 0.07). Bcl-2 expression was higher in SCLC patients (46.3% versus 28.3%, p = 0.03) and was associated with worse prognosis in EPNEC (median OS 8.0 months versus 14.7 months; HR 0.47, p = 0.02).

**Conclusion:**

EPNEC patients presented inferior RR to platinum-based chemotherapy than SCLC but tended to live longer. Neither ERCC1, Lin28, or Ki-67 were prognostic or predictive for RR in EPNEC or SCLC. High Bcl-2 expression was associated with poor prognosis in EPNEC patients.

## Introduction

Neuroendocrine tumours are rare, but their incidence has increased over the past years [1]. While the Grade 1 (Ki-67 ≤ 2% and < 2 mitoses/10 HPF) and Grade 2 (Ki-67 3% to 20% and 2 to 20 mitoses/10 HPF) neuroendocrine tumours (NET) treatment is mainly based on molecular targeted therapy, extrapulmonary neuroendocrine carcinomas (Grade 3: Ki-67 > 20% or > 20 mitoses/10 HPF) [2] are treated with platinum-based chemotherapy [3, 4] extrapolating the data from small cell lung cancer (SCLC). However, despite sharing similar histopathological features, disease behaviour and treatment outcomes may differ between these two diseases.

In a retrospective study, patients with extrapulmonary neuroendocrine carcinomas (EPNEC) treated with platinum-based chemotherapy responded less but lived longer than those with extensive SCLC: response rate (RR) was 30.8% and 77.8% (p<0.001) and median overall survivals (OS) were 13.6 and 9.2 months, respectively (p = 0.07) [5]. One possible explanation is that EPNEC and SCLC may harbour distinctive biology with different biomarkers playing unique roles in each carcinogenesis. In a retrospective analysis [6], for example, Bcl-2 was overexpressed in 33% of EPNEC, while literature data [7] show that Bcl-2 overexpression is seen in more than 70% of SCLC.

Based on data from studies in other cancers, certain biomarkers could be potentially associated with platinum resistance in EPNEC and SCLC such as ERCC1 (Excision repair cross-complementation group 1), Lin28a, Bcl-2 (B-cell lymphoma 2) and Ki-67. ERCC1 has been related to platinum resistance in other solid tumours [8-11]. Bcl-2, a anti-apoptotic protein [12], and Lin28a, a micro-RNA let7 regulator, have been both associated with higher proliferation rates and less cellular differentiation [13] and could influence both prognosis and response to chemotherapy. However, little is known about the frequency of expression, prognostic, and predictive effects of these proteins in EPNEC or SCLC.

Based on the hypothesis that SCLC and EPNEC are distinct entities, our objective was to compare the frequency of expression of ERCC1, Lin28a, and Bcl-2 and their impact on survival and treatment response to platinum-based chemotherapy in patients with advanced/metastatic EPNEC or SCLC. We also evaluated the predictive effect of the cellular proliferation marker Ki-67 given its clinical importance in G3 tumours [3].

## Materials and Methods

### Study design and eligibility criteria

This is a retrospective multicentre cohort study of EPNEC and SCLC patients treated with first-line platinum-based chemotherapy at three Brazilian cancer centres (Hospital Sírio-Libanês, Instituto do Câncer do Estado de São Paulo and Instituto do Câncer do Ceará) between 2006 and 2014. Consecutive patients were identified from the hospitals’ administrative databases, and all clinical information was obtained from electronic medical records.

Eligible criteria were a histologically confirmed diagnosis of advanced EPNEC or SCLC, Ki-67 proliferative index >20% or mitotic index >20 mitoses/10 HPF for EPNEC, availability of tumour samples for biomarkers analyses by immunohistochemistry (IHC), radiological measurable disease, completed at least one cycle of platinum-based chemotherapy, and availability of radiological exams before and after treatment for RR analysis. The study was approved by the local Institutional Review Board (IRB) and all patients who were alive signed the informed consent forms.

Patients evaluated had been treated with cisplatin 25 mg/m^2^ and etoposide 100 mg/m^2^ intravenously (IV) on days 1, 2, and 3 or irinotecan 60 mg/m^2^ plus cisplatin 30 mg/m^2^ IV on days 1 and 8, both repeated with a three-week interval [4]. Also, some EPNEC patients had received the CapOx regimen with oxaliplatin 130 mg/m^2^ IV on day 1 and capecitabine 1000 mg/m^2^ orally twice per day from day 1 to day 14 repeated at three week intervals [14], and those with renal dysfunction had received carboplatin in substitution for cisplatin.

Our primary endpoint was RR according to response evaluation criteria in solid tumors (RECIST) (v1.1) after at least one cycle of treatment. The same radiologist (B.G.) reviewed all digital images to determine radiological response.

### Tumour samples and biomarkers analyses

A NET specialised pathologist (R.S.S.M.), unaware of clinical data, reviewed all tumour samples to determine the differentiation grade and Ki-67 index using the hotspot method. Patients whose tumour material did not have adequate quality for IHC analysis and those re-classified as G2 NET were excluded. Pathology specimens from either the primary tumour or metastasis were utilised.

Immunohistochemistry (IHC) staining of formalin-fixed, paraffin-embedded tumour tissues obtained prior to chemotherapy initiation was performed using monoclonal antibodies against ERCC1 (clone SP68: Spring, cat# M3684 Pleasanton, CA, EUA; working dilution 1:200), Bcl-2 (clone 124: Cell Marque, cat# 226M-96, Rockin, CA, EUA; working dilution 1:400), Lin28a (clone EPR4640: Epitomics, cat# 3533-1, Burligame, CA, EUA; working dilution 1:50), and Ki-67 (MIB1 antibody). The positive controls were those known to be positive for the specific antibody or lysates of transfected cells that are commercially sold for this purpose and negative control was performed by omission of the primary antibody (Table 1). A final semiquantitative IHC score (H-score) was calculated multiplying the intensity of staining (scale of 0 to 3, being 0 = no staining, and 3 = strong staining) by the percentage of positive cells (scale of 0–100). The H-score ranges from 0–300 and for all biomarkers the protein expression was considered positive (high expression) when H-score ≥ 200. For each IHC analyses, positive, and negative controls were used.

### Statistical analyses

Because of the exploratory nature of our study, biomarkers were analysed as both categorical and continuous variables. Associations between each biomarker expression and RR, and the comparison between RR among different groups (EPNEC and SLCL; well and poorly differentiated EPNEC) were evaluated using Chi-square or Fisher’s exact test for categorical variables, and t-test or univariate logistic regression used for continuous variables.

The analysis of the Ki-67 as a categorical variable considered the cutoff value of 55% (Ki-67 <55% and ≥ 55% Ki-67) [3].

OS was defined as the time from the date of the first cycle of platinum-based chemotherapy to death from any cause, and it was estimated using the Kaplan-Meier method. The time-to-event variables were compared by log-rank between high expression and low expression for each biomarker, between patients with EPNEC and SCLC, and between well-differentiated and poorly differentiated EPNEC. Cox regression analysis was used to evaluate biomarkers expression (continuous variables) and OS. Data on survivors were censored at the last follow-up. Two-tailed p values of < 0.05 were considered statistically significant. All analyses were performed using the SPSS statistical software programme package (SPSS version 21.0 for Mac).

## Results

Between July, 2006 and July, 2014, we identified 201 SCLC patients and 125 EPNEC patients with unresectable advanced or metastatic disease treated with platinum-based chemotherapy. Of these, 82 SCLC patients and 60 EPNEC were eligible for the study and had their tumour tissues analysed for biomarkers IHC expression. Four patients in EPNEC group and 14 in SCLC group did not have adequate radiologic evaluation according to the RECIST 1.1, and four EPNEC patients and six SCLC patients were initially diagnosed with stage III (locally advanced, unresectable) disease. Finally, 56 patients in EPNEC group and 68 in SCLC group were eligible for response evaluation and 56 in EPNEC group and 76 in SCLC were eligible for OS analysis.

Baseline clinical characteristics were similar in both groups except for age and smoking which were more common in the SCLC group. Some histological features were different between the groups, given that large cell histology and well-differentiated tumours were restricted to the EPNEC group (Table 2).

### EPNEC and SCLC: response rate and overall survival

**Figure 1.**
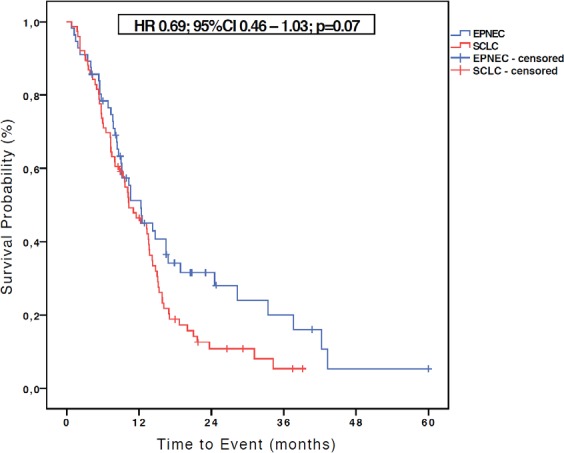
Kaplan-Meier curves of OS for EPNEC versus SCLC.

With a median follow-up of 10.5 months (1.2–60 months) for patients with EPNEC and of 10.3 months (1.7–39.1 months) for those with SCLC, the median OS in the SCLC (N = 76) group was 10.3 months, while for EPNEC (N = 56) it was 11.1 months (HR 0.69, 95% CI 0.46–1.03; p = 0.07) as shown in the Figure 1. At the time of OS analysis, 12 (20%) EPNEC patients and 7 (8.5%) SCLC patients were alive.

Of the 56 patients in EPNEC group with available radiologic evaluation, 25 (44.6%) experienced objective response to platinum-based chemotherapy; in the SCLC group (68 patients evaluable) the CR or PR was observed to be 59 (RR = 86.8%) patients (p<0.001). This difference in RR remained significant regardless of the type of platinum utilised: cisplatin and etoposide led to CR/PR of 46.7% (14 out of 30) of EPNEC patients and in 88.1% (59 out of 67) of those with SCLC (p<0.001). Likewise, the RR in the EPNEC group (N = 56) persisted inferior when we analysed the subgroups of gastrointestinal EPNEC (N = 33/58.9%), small cell histology (N = 46/82.1%) or poorly differentiated tumours (N = 43/76.8%). The RR in these three subgroups were 45.5%, 43.5%, and 48.9% respectively, (p<0.001 in all analyses when compared with RR among SCLC patients).

### Biomarkers analyses

The median Ki-67 expression was 60% (range: 7–100%) among SCLC patients and 50% (20–95%) in those with EPNEC (p = 0.86). Forty-seven (78.3%) out of 60 EPNEC patients and 70 (85.4%) out of 82 SCLC patients had tumours with high expression of ERCC1 (H-score ≥ 200) (p = 0.28). Also, the median and mean ERCC1 H-scores were 300 (range 0-300) and 260.24 (SD: ± 68.31) for SCLC and 300 (range 0–300) and 239.58 (SD: ± 88.65) for EPNEC.

Regarding Lin28a expression, 35 (59.3%) patients with EPNEC (N = 59; in one patient the material was not adequate for Lin28a analysis) and 35 (42.7%) of those with SCLC (N = 82) had tumours which expressed an H-score ≥ 200 (p = 0.05). The median and mean Lin28a H-scores were 155 (range 0–300) and 155.55 (SD: ± 112.59) for SCLC and 200 (range 0–300) and 190.51 (SD: ± 102.44) for EPNEC.

Bcl-2 had significantly higher expression in the SCLC group (38 out of 82; 46.3%) in comparison with EPNEC patients (17 out of 60; 28.3%) (p = 0.03). The median and mean H-scores were 180 (range 0-300) and 165.91 (SD: ± 110) in the SCLC group and 31.25 (range 0-300) and 98.04 (SD: ± 119) in the EPNEC patients.

We found no association between each biomarker expression and RR in either EPNEC or SCLC patients when we tested their IHC expression as either categorical (Table 3) or continuous variables (Table 4).

**Figure 2.**
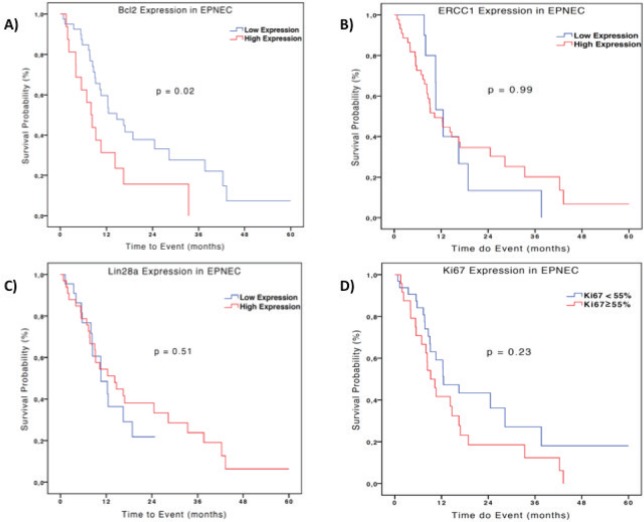
Kaplan-Meier curves of OS of 56 patients with EPNEC according to IHC expression (high expression: H-score ≥200) Bcl-2 (A), ERCC1 (B), Lin28a (C), and Ki-67 (D). Difference between the curves was evaluated by the log-rank test.

In terms of prognosis, there was no difference between the median OS according to ERCC1 expression in the EPNEC group: 10.3 months for low versus 12.5 months for high expression (HR 1.0; 95% CI 0.458–2.185, p = 0.99). For Lin28a high expression patients had a median OS of 10.6 months versus 14.2 months (HR 1.26; 95% CI 0.64–2.45; p = 0.51) for those with low expression. EPNEC patients with Ki-67 < 55% presented median survival of 14.2 months versus 10.3 months for those with Ki-67 ≥ 55% (HR 0.68, 95% CI 0.35–1.29; p = 0.23). Bcl-2 high expression was significantly associated with inferior OS when compared with the low expression (8.0 months and 14.7 months respectively; HR 0.47, 95% CI 0.24–0.91; p = 0.02)–Figure 2. Cox regression analysis which was utilised to evaluate the biomarkers as continuous variables showed similar results, maintaining a significant but marginal association between Bcl-2 expression and OS in EPNEC patients (HR 1.003; CI 95% 1.0005–1.006; p = 0.02). The other biomarkers were not significantly associated with OS (ERCC1: HR 1.0, 95% CI 0.99–1.01, p = 0.24; Lin28a: HR 1.0, 95% CI 0.99–1.0, p = 0.56; Ki-67: HR 1.01, 95% CI 0.99–1.02, p = 0.11).

**Figure 3.**
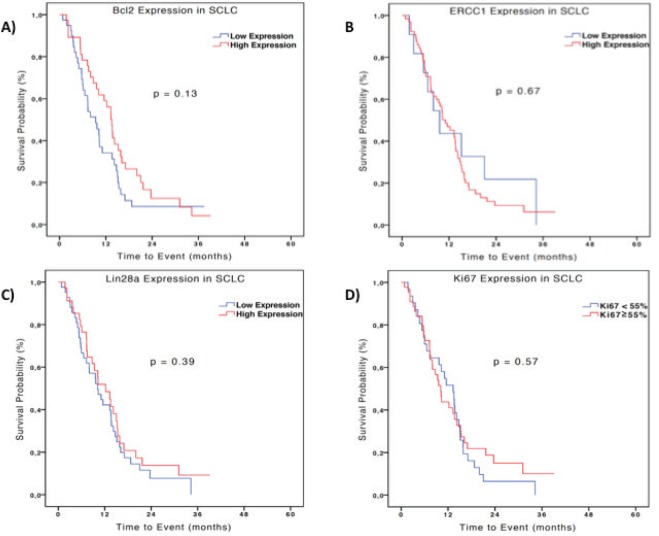
Kaplan-Meier curves of OS of 76 patients with SCLC according to IHC expression (high expression: H-score ≥ 200) Bcl-2 (A), ERCC1 (B), Lin28a (C) and Ki-67 (D). Difference between the curves was evaluated by the log-rank test.

In the SCLC group, the median OS was 9.6 and 11.1 months in the groups with low and high expression ERCC1 respectively (HR 0.86; 95%CI 0.42–1.74; p=0.67). The analysis of Bcl-2 showed an opposite tendency to what was seen in patients with EPNEC with an OS numerically lower in patients with low expression (9.3 months) versus high expression (13.5 months) (HR 1.45; 95% CI 0.89–2.36; p = 0.13). Also there was no difference in OS between patients with low expression (10.1 months) and high expression of Lin28a (12.2 months) (HR 1.24, 95% CI 0.76–2.02; p=0.39), or between patients with Ki-67 <55% and with Ki-67 ≥ 55% respectively (13.5 months versus 10.1 months [HR 1.15; 95% CI 0.70–1,88, p = 0.57])–Figure 3. Analysing the biomarkers as continuous variables, there was no significant association between OS and expressions of ERCC1 (HR 1.0; 95% CI 0.99–1.0; p = 0.34), Lin28a (HR 0.99; 95% CI 0.99–1.0; p = 0.26), Ki-67 (HR 0.99; 95% CI 0.98-1.0; p = 0.18) or Bcl-2 (HR 0.99; 95% CI 0.99–1.0; p = 0.05) in patients with SCLC.

### Well-differentiated and poorly differentiated EPNEC: response rate and overall survival

**Figure 4.**
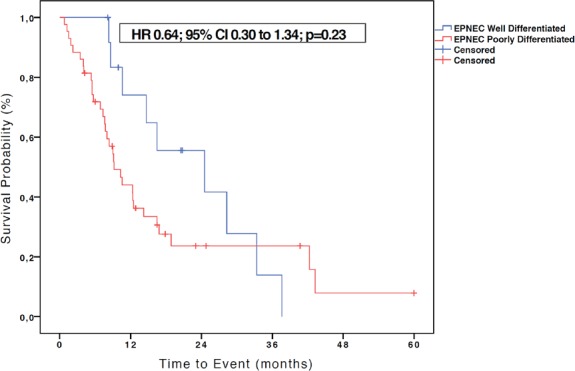
Kaplan-Meier curves of OS of 56 patients with EPNEC available to survival analysis according to the degree of differentiation (well differentiated and poorly differentiated). The difference between the curves was evaluated by the log-rank method.

Among the 60 patients with metastatic EPNEC, 13 (21.7%) had well-differentiated histology and 47 (78.3%) were classified as poorly differentiated. The median OS in the group of well-differentiated EPNEC was 24.5 months versus 9.2 months for the poorly differentiated histology group (HR 0.64; 95% CI 0.30–1.34; p = 0.23)–Figure 4.

From the 56 patients with EPNEC eligible for RR analysis, 13 (23.2%) were classified as well-differentiated carcinomas and 43 (76.8%) as poorly differentiated. RR was similar between the subgroups: 38.5% for well-differentiated versus 39.5% for poorly differentiated EPNEC (p = 0.94).

The high expression of ERCC1, Bcl-2, and Lin28 were similar in both well (N = 13) and poorly differentiated group (N = 47), respectively: nine (69.2%) and 38 (80.1%) patients (p = 0.37); three (23.1%) and 14 (29.8%) patients (p = 0.63); and six (46.1%) and 29 (61.7%) patients (p = 0.46). However, Ki-67 index was ≥ 55% in 28 (59.6%) poorly differentiated and only in two (15.4%) well-differentiated EPNEC (p = 0.048).

## Discussion

There are no randomised trials that support the use of systemic therapy for patients with advanced EPNEC. While most patients with metastatic EPNEC are treated with platinum-based chemotherapy, as a data extrapolation from patients with SCLC, disease behaviour and treatment outcomes for these entities appear to differ considerably. Studies investigating predictive and prognosis biomarkers could contribute to a better understanding of such differences.

In this retrospective study, we found that while patients with metastatic EPNEC had inferior RR to platinum-based chemotherapy (p<0.001), they tended to live longer in comparison with SCLC patients (p = 0.07). Most patients with SCLC and EPNEC presented high expression of ERCC1 and Lin28, but Bcl-2 expression was more frequent in SCLC patients (p = 0.03). However, neither ERCC1, Bcl-2, Lin28a, or Ki-67 high expression were predictive of RR in SCLC or EPNEC. When evaluating the influence of the molecular markers’ expression on OS, we observed that only Bcl-2 hyperexpression was associated with worse survival in patients with EPNEC (p = 0.02). In comparison with poorly differentiated EPNEC, the subgroup of patients with well-differentiated tumours presented comparable frequencies of ERCC1, Bcl-2, and Lin28 IHC expression, similar RR (p = 0.94), but higher Ki-67 index (p = 0.048) and numerically longer OS (p = 0.23).

We are unaware of studies that have reported the expression of ERCC1 and Lin28a in EPNEC, but there are few publications available on the Bcl-2 expression. The reasons why the selected biomarkers had no influence on response or survival are unknown. One possible issue is the measurement of ERCC1 expression. This is because there is no consensus if immunohistochemistry is the best method and which antibody to use, or if PCR should be the preferred method. The study published by Friboulet *et al* [15] with non-small cell lung cancer patients, where he unsuccessfully tried to revalidate the data published in 2006 [16], he showed that there are four known isoforms of ERCC1 with only one being active, and it was not possible to determine which form is expressed by immunohistochemistry. Of note, this study [15] was published in 2013 and our protocol was written in 2011, and until then there were no data that called into question the use of immunohistochemistry to analyse the expression of ERCC1. Added to this, given the lack of guidelines on how to measure ERCC1, we decided to use IHC because it is an inexpensive and quick method to screen protein expression. However, the overexpression of nonfunctioning isoforms may result in a false-positive expression and could lead to a bias when interpreting data.

In our patients, higher expression of Bcl-2 was found in SCLC compared to EPNEC (46.3% versus 28.3%, respectively; p = 0.03) which is consistent with the data shown by Brenner B *et al* [7]. Because the Bcl-2 protein has anti-apoptotic function, its higher expression in SCLC patients could somehow lead to chemoresistance and worse OS. However, we were unable to demonstrate an association between Bcl-2 expression and either RR or OS in patients with SCLC. On the other hand, for the patients with EPNEC treated with platinum-based chemotherapy, low Bcl-2 expression (H-score <200) was associated with improved OS in comparison to high expression (p=0.02) with a numerical gain of 6.7 months. Although statistically significant, we consider the HR of 1.003 to be clinically irrelevant (HR>1, 95% CI 1.0005–1.006), representing a 0.3% risk of death when there is Bcl-2 high expression. This same association had already been shown in the literature for lung NET, including typical carcinoid, atypical carcinoid, and large cell carcinoma [17]. In this study, Bcl-2 was highly expressed in 18.7% (N = 3/16) patients with typical carcinoid, in none (N = 0/5) with atypical carcinoid, in 89.6% (N=26/29) with large cell carcinoma, and 90.1% (N = 64/71) of those with SCLC. Similar to our data, this study showed that Bcl-2 higher expression was associated with poor cell differentiation but also failed to demonstrate the influence of Bcl-2 expression in the outcomes in SCLC patients. The difference in the levels of Bcl-2 overexpression between our data and some other studies [7] could be because of the lack of standardisation in the cutoffs to define high and low expression. Our findings suggest that Bcl-2 may be prognostic in EPNEC but not in SCLC which already harbours dismal prognosis.

Studies in other solid tumours have linked the high expression of Lin28a to platinum-resistance and less differentiated histology [18, 19]. Although not statistically significant, we observed that Lin28a was more commonly expressed in poorly differentiated (61.7%) versus well-differentiated (46.1%) EPNEC (p = 0.46). In contrast to studies with another tumours [18], both EPNEC and SCLC patients with Lin28a hyperexpression presented OS numerically longer than those with lower expression (p = 0.51 and p = 0.39 respectively), but it was not statistically significant, and for this reason it is not possible to determine its relevance in EPNEC or SCLC.

Our data showed that the Ki-67 expression was not predictive of response to platinum-based chemotherapy in patients with EPNEC (p = 0.88). This finding contrasts with the data from the Nordic study [3] where Ki-67 >55% was associated with higher RR. It is possible that this results are from a smaller sample. Similarly to the Nordic study, the EPNEC group with Ki-67<55% had a trend towards higher OS compared to the group with ≥55% (14.2 versus 10.3 months respectively; p = 0.23). However, in an equally large series of 294 patients with EPNEC [20], Ki-67≥55% was not associated with worse OS. We think that it is probably inadequate to rely solely on Ki-67 to dictate prognosis in NET. It is possible that such discrepancies in Ki-67 across studies may reflect different proportions of patients with well and poorly differentiated EPNEC. For example, a retrospective series found longer OS and lower RR for well-differentiated EPNEC patients when compared with those with poorly differentiated NEC [21, 22]. These findings point to tumour heterogeneity within the G3 group. However, in our study although the OS was longer for well-differentiated EPNEC, the RR was similar between well and poorly differentiated EPNEC. In our opinion other factors beyond Ki-67 and cell differentiation such as primary sites and unknown molecular heterogeneity may influence treatment outcomes.

We also observed that while survival was similar (p = 0.07), the RR to chemotherapy was higher among patients with SCLC when compared to patients with EPNEC (p<0.001), even after analysing separately those with poorly differentiated tumours, small cell histology and primary tumour in the gastrointestinal tract or only those who received chemotherapy with cisplatin and etoposide in an attempt to standardise the EPNEC group. These data are consistent with what was published by Terashima *et al* [5], showing that the patients with SCLC have higher RR to platinum-based chemotherapy when compared with EPNEC but without significant difference in OS. This finding supports our hypothesis that the two diseases have distinct carcinogenesis pathways and molecular specific drivers. For example, less than half of EPNEC patients had a history of smoking compared with almost all SCLC patients. Furthermore, NEC are heterogeneous as reflected by the presence of well and poorly differentiated histologies within the G3 group. Hence, it is possible that there are different biomarkers expressions depending on the site of biopsy, tumour primary site, or cell differentiation which would result in different expression rates and could influence the analysed data. Following this hypothesis, a recent retrospective study [23] showed that mutual genetic alterations in retinoblastoma protein (RB1) and tumour protein 53 (TP53) are more common in SCLC than in EPNEC, and that this differs according to the EPNEC primary site. Besides that, another study [24] suggested an alternative pathway for SCLC where it may originate from non-neuroendocrine cancer stem cells that acquire neuroendocrine differentiation through inactivating NOTCH (neurogenic locus notch homolog) mutations, ASCL1 (Achaete-scute homolog 1) overexpression and additionally a bi-allelic loss of TP53 and RB1.

Our study has some limitations. As it is retrospective, external validation should be made with caution. In addition, the variety of primary sites in EPNEC, utilisation of tumour tissues from either primary or metastatic sites, the small number of patients, and the lack of standardisation to measure the IHC expressions of biomarkers have also to be taken into account when interpreting our results. Despite the limiting factors, all patients had their tumour specimens centrally evaluated by a single pathologist and all images were analysed using RECIST 1.1 by the same radiologist. Given the originality of our study, we think our results add value to future studies of NEC.

New molecular agents have not been formally tested in clinical trials of EPNEC. Ongoing trials with everolimus will be completed soon, and it might shed some light on the role of mTOR pathway in this disease. In the era of targeted therapy, the studies of predictive factors are of prime importance to select patients who are more likely to benefit and to reduce treatment cost. Although we could not identify any predictor of RR to chemotherapy among the selected biomarkers, our results demonstrated that high expressed Bcl-2 was associated with shorter OS in patients with advanced EPNEC. This finding could be further explored and if more evidence emerges, Bcl-2 could be a potential new therapeutic target in G3 NEC, although anti-Bcl-2 therapies have not been successfully developed so far [25].

## Conclusions

In conclusion, the biomarkers ERCC1, Bcl-2, Lin28a, and Ki-67 were not associated with RR to platinum-based chemotherapy in both EPNEC and SCLC. Bcl-2, when highly expressed, was associated with poor OS in EPNEC patients. Even though SCLC and EPNEC are treated similarly in this cohort, significant differences in RR and Bcl-2 expression were noted. When compared with SCLC, EPNEC patients had lower response to platinum-based chemotherapy but presented numerically longer survival. Large collaborative efforts, multicentre prospective databases, and exploratory phase II clinical trials, including proof-of-concept trials are urged in order to help us understand and better treat patients with EPNEC.

## Conflicts of interest

All authors declare that we have no conflicts of interest in the authorship or publication of this contribution.

## Figures and Tables

**Table 1.** Positive controls used in immunohistochemistry.

Control Sample	Antibody	Clones	Titration	Manufacturers
**Tonsil**	Bcl2	124	1:400	Cell Marque, catalog 226M-96, Rocklin, CA, EUA
**Colon carcinoma**	ERCC1	SP68	1:200	Spring, catalog M3684, Pleasanton, CA, EUA
**Testicle**	Lin28a	EPR4640	1:50	Epitomics, catalog 3533-1, Burligame, CA, EUA
**Tonsil**	Ki-67	MIB-1	1:1000	DAKO, Glostrup, Denmark

**Table 2.** Patients and treatment characteristics.

	EPNEC (N = 60)	SCLC (N = 82)	*p*
**Age in years: median (range)**	55.5 (23–81)	59 (35–81)	0.03
**Gender** **Male** **Female**	34 (56.7%) 26 (43.3%)	48 (58.5%) 34 (41.5%)	0.82
**ECOG** **PS 0/1** **PS 2/3**	45 (75%) 15 (25%)	55 (67.1%) 27 (32.9%)	0.31
**Smoking history**	28 (46.7%)	81 (98.8%)	<0.001
**Staging** **III** **IV**	4 (6.7%) 56 (93.3%)	6 (7.3%) 76 (92.7%)	0.88
**Ki67% (median, range)**	50% (20–95%)	60% (7–100%)	0.86
**Histology** **Small cell** **Large cell**	49 (81.7%) 11 (18.3%)	82 (100%) —	<0.001
**Tumour cell differentiation** **Well-differentiated** **Poorly differentiated**	13 (21.7%) 47 (78.3%)	— 82 (100%)	<0.001
**Type of first-line chemotherapy**			
**- Platinum + etoposide**	32 (53.3%)	80 (97.4%)	<0.001
**- Platinum + irinotecan**	20 (33.4%)	—	
**- Capecitabine + oxaliplatin**	8 (13.3%)	—	
**- Cisplatin + gemcitabine**	—	1 (1.2%)	
**- Carboplatin + paclitaxel**	—	1 (1.2%)	
**Number of cycles (median, range)**	5 (1–10)	5 (1–6)	0.76
**Patients who received second line chemotherapy**	32 (53.3%)	46 (56.1%)	0.70
**Primary sites** Pancreas Colorectal Stomach Oesophageal Cervical Bladder Nasopharyngeal Nasal Cavity Duodenum Hypopharyngeal Gallbladder Prostate Unknown primary site	16 (26.7%) 7 (11.7%) 5 (8.3%) 4 (6.7%) 4 (6.7%) 3 (5%) 3 (5%) 2 (3.3%) 2 (3.3%) 1 (1.7%) 1 (1.7%) 1 (1.7%) 11 (18.3%)	— — — — — — — — — — — — —	

**Table 3.** Association between biomarkers expression analysed as categorical variables and response rate (RR) to chemotherapy.

Biomarkers	EPNEC (N = 56 patients evaluable) P-value (X2/Fisher)	SCLC (N = 68 patients evaluable) P-value (X2/Fisher)
ERCC1 (RR in High versus low expression)	44.2% versus 46.2% (P = 0.90)	86% versus 90.9% (P = 1.000)
Bcl-2 (RR in High versus low expression)	37.5% versus 47.5% (P = 0.50)	84.8% versus 88.6% (P = 0.730)
Lin28a (RR in High versus low expression)	46.9% versus 43.5% (P = 0.80)	82.8% versus 89.7% (P = 0.481)
Ki-67 (≥55% versus <55%)	46.4% versus 44.4% (P = 0.88)	87.2% versus 85.7% (P = 1.000)

**Table 4.** Association between biomarkers expression analysed as continuous variable and response rate to chemotherapy.

SCLC (N = 68 evaluable for response)			
**Biomarkers**	**Odds Ratio**	**CI (95%)**	**p-value**
ERCC1	0.994	[0.982; 1.008]	0.40
Bcl-2	0.996	[0.989; 1.003]	0.27
Lin28a	1.001	[0.995; 1.007]	0.86
Ki-67	1.011	[0.983; 1.04]	0.45
**EPNEC (N = 56 evaluable for response)**			
**Biomarkers**	**Odds Ratio**	**CI (95%)**	**p-value**
ERCC1	1.002	[0.996; 1.008]	0.61
Bcl-2	0.999	[0.995; 1.004]	0.70
Lin28a	1.0005	[0.995; 1.006]	0.86
Ki-67	1.002	[0.981; 1.023]	0.86
